# Bis(4-hy­droxy-3-meth­oxy­benzaldehyde 4-phenyl­thio­semicarbazonato-*N* ^1^,*S*)nickel(II)

**DOI:** 10.1107/S1600536814009027

**Published:** 2014-04-30

**Authors:** Adriano Bof de Oliveira, Bárbara Regina Santos Feitosa, Christian Näther, Inke Jess

**Affiliations:** a Departamento de Química, Universidade Federal de Sergipe, Av. Marechal Rondon s/n, Campus, 49100-000 São Cristóvão-SE, Brazil; b Institut für Anorganische Chemie, Christian-Albrechts-Universität zu Kiel, Max-Eyth Strasse 2, D-24118 Kiel, Germany

## Abstract

In the title compound, [Ni(C_15_H_14_N_3_O_2_S)_2_], the Ni^II^ atom lies on a center of symmetry. The deprotonated ligands act as *N*,*S*-donors, forming five-membered metalla-rings. The Ni^II^ atom is four-coordinated in a slightly distorted square-planar environment. In the crystal, the discrete complex mol­ecules are linked by weak N—H⋯O hydrogen bonds, generating chains along [110]. The chains are further connected *via* weak O—H⋯N inter­actions into a layered network extending parallel to (001).

## Related literature   

For the crystal structure of the ligand, see: Oliveira *et al.* (2013 ▶). For the crystal structure of a similar complex, see: Akinchan & Abram (2000 ▶). For the coordination chemistry of thio­semicarbazone compounds, see: Lobana *et al.* (2009 ▶).
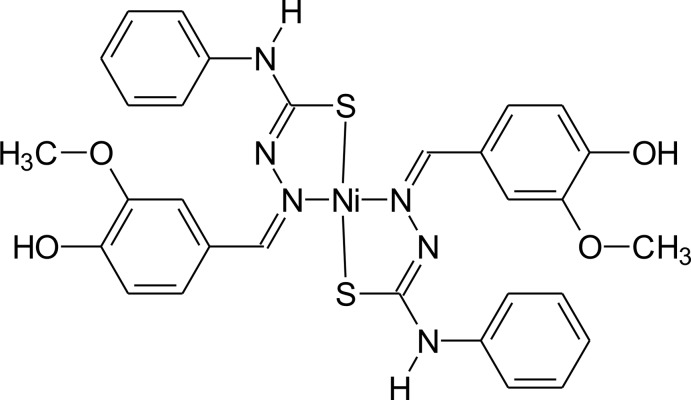


## Experimental   

### 

#### Crystal data  

[Ni(C_15_H_14_N_3_O_2_S)_2_]
*M* *_r_* = 659.41
Triclinic, 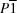

*a* = 6.8080 (4) Å
*b* = 7.5569 (4) Å
*c* = 14.3902 (8) Å
α = 98.514 (4)°
β = 92.062 (5)°
γ = 102.698 (5)°
*V* = 712.47 (7) Å^3^
*Z* = 1
Mo *K*α radiation
μ = 0.88 mm^−1^
*T* = 200 K
0.12 × 0.08 × 0.04 mm

#### Data collection  

Stoe IPDS-1 diffractometer
Absorption correction: numerical (*X-SHAPE* and *X-RED32*; Stoe & Cie, 2008 ▶) *T*_min_ = 0.800, *T*_max_ = 0.936
3117 measured reflections
2539 independent reflections
2539 reflections with *I* > 2σ(*I*)
*R*_int_ = 0.033

#### Refinement  

*R*[*F* ^2^ > 2σ(*F* ^2^)] = 0.030
*wR*(*F* ^2^) = 0.080
*S* = 1.06
3117 reflections
206 parameters
H atoms treated by a mixture of independent and constrained refinement
Δρ_max_ = 0.33 e Å^−3^
Δρ_min_ = −0.19 e Å^−3^

### 

Data collection: *X-AREA* (Stoe & Cie, 2008 ▶); cell refinement: *X-AREA*; data reduction: *X-RED32* (Stoe & Cie, 2008 ▶); program(s) used to solve structure: *SHELXS97* (Sheldrick, 2008 ▶); program(s) used to refine structure: *SHELXL97* (Sheldrick, 2008 ▶); molecular graphics: *DIAMOND* (Brandenburg, 2006 ▶); software used to prepare material for publication: *publCIF* (Westrip, 2010 ▶).

## Supplementary Material

Crystal structure: contains datablock(s) I. DOI: 10.1107/S1600536814009027/lr2126sup1.cif

Structure factors: contains datablock(s) I. DOI: 10.1107/S1600536814009027/lr2126Isup2.hkl

CCDC reference: 998671

Additional supporting information:  crystallographic information; 3D view; checkCIF report

## Figures and Tables

**Table 1:** Hydrogen-bond geometry (Å, °)

*D*—H⋯*A*	*D*—H	H⋯*A*	*D*⋯*A*	*D*—H⋯*A*
N1—H1*N*1⋯O1^i^	0.81 (3)	2.37 (3)	3.122 (2)	154 (2)
O1—H1*O*1⋯N2^ii^	0.84	2.54	3.131 (2)	129

Symmetry codes: (i) 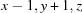; (ii) 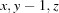.

**Fig. 1.**
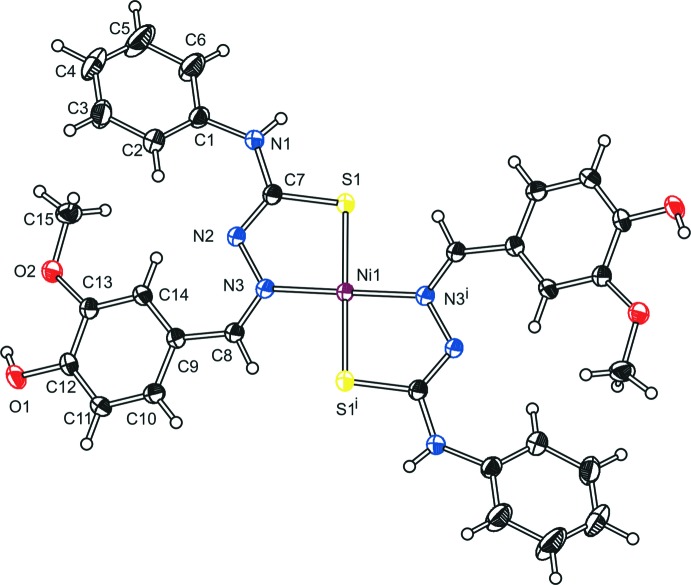
: The molecular structure of the title compound with displacement ellipsoids drawn at the 40% probability level. Symmetry code for the generation of equivalent atoms: (i)-x + 1,-y + 2,-z.

**Fig. 2.**
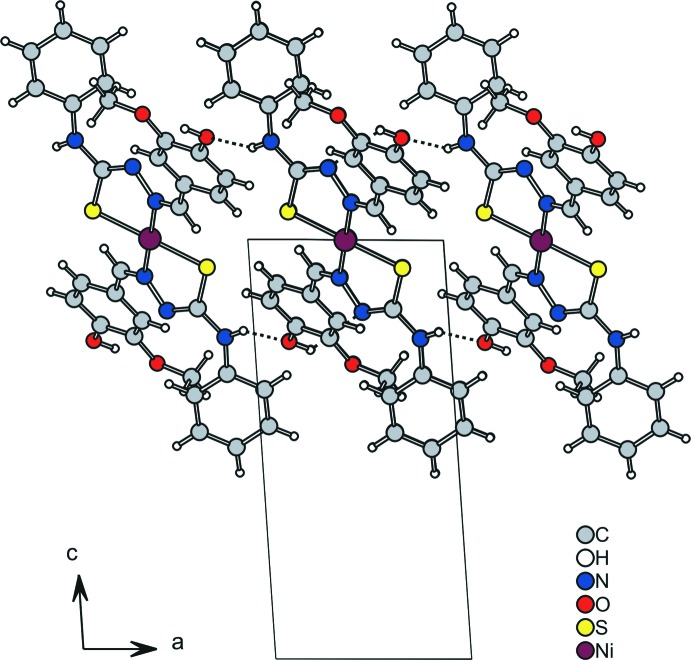
: Crystal structure of the title compound with view along the b-axis. The hydrogen interactions are shown as dashed lines.

## supplementary crystallographic information

### 2.1. Synthesis and crystallization

Starting materials were commercially available and were used without further purification. 4-Hy­droxy-3-meth­oxy­benzaldehyde 4-phenyl­thio­semicarbazone was dissolved in THF (2 mmol/40 ml) with stirring maintained for 30 min, while the solution turns yellow. A solution of nickel acetate tetra­hydrate (1 mmol/40 ml) in THF was added under continuous stirring. After 3 h the solvent was removed and the solid redissolved in methanol. Crystals suitable for X-ray diffraction were obtained by the slow evaporation of the solvent.

### 2.2. Refinement

Crystal data, data collection and structure refinement details are summarized in Table 1. All non-hydrogen atoms were refined anisotropic. Most C—H atoms were positioned with idealized geometry (methyl and O—H atoms allowed to rotate but no to tip) and were refined isotropic with *U*_iso_(H) = 1.2 *U*_eq_(C, N) (1.5 for methyl and O—H atoms) using a riding model. The H atoms attached to N1 and C8 were refined with varying coordinates and varying isotropic displacement parameters.

### 3. Results and discussion

Thio­semicarbazone derivatives are *N*,*S*-donors with a wide range of coordination modes (Lobana *et al.*, 2009). As part of our inter­est on the coordination chemistry of thio­semicarbazone ligands, we report herein the synthesis and the crystal structure of a new Ni^II^ complex with the 4-hy­droxy-3-meth­oxy­benzaldehyde 4-phenyl­thio­semicarbazone.

The Ni^II^ atoms are four-coordinated in a slightly distorted planar environment by two bidentate deprotonated ligands forming discrete complexes. The asymmetric unit consists of one Ni^II^ cation that is located on a centre of inversion and one anionic ligand that occupies a general position (Fig. 1). During complex formation signficant structural changes of the N–N–C–S fragment are observed. For the uncoordinated 4-hy­droxy-3-meth­oxy­benzaldehyde 4-phenyl­thio­semicarbazone ligand the N–N, N–C and C–S bond distances amount to 1.3792 (17) Å, 1.3404 (19) Å and 1.6962 (15) Å. The distances indicate the double bond character for the N–N and C–S bonds, and the single bond character for the N–C bond (Oliveira *et al.*, 2013).

For the title compound, the acidic hydrogen of the hydrazine fragment is lost and the negative charge is delocalized over the N–N–C–S fragment. Therefore, for the coordinated ligand the N–N, N–C and C–S bond distances amount to 1.407 (4) Å, 1.306 (3) Å and 1.732 (4) Å. Similar values are found in the literature for the bis­(4-hy­droxy-3-meth­oxy­benzaldehyde thio­semicarbazonato-*N*^1^,*S*)nickel(II) complex: 1.401 (3) Å, 1.317 (3) Å and 1.726 (3) Å (Akinchan & Abram, 2000). The N–C bond distances indicate a considerable double bond character, while the N–N and C–S bond distances are consistent with an increased single bond character.

The ligands are coordinated to the metal as *N*,*S*-donors (Fig. 1), building a slightly distorted planar environment, typical for low spin, strong field and *d*^8^ electronic configuration with Jahn-Teller effect. The maximal deviation from the least squares plane through all non-hydrogen atoms for the Ni1/C7/N2/N3/S1 ring amounts to 0.2373 (15) Å for N3. Additionally, the dihedral angle between the two aromatic rings of the ligands is 42.270 (68)°, showing that they are not planar (Fig. 1).

The molecules are linked into chains along the *a-b*-direction forming a H-bonded coordination polymer (Fig. 2). The crystal packing is stabilized by inter­molecular N—H···O and O—H···N hydrogen bonding (Table 1).

### Crystal data

**Table:**

[Ni(C_15_H_14_N_3_O_2_S)_2_]	*V* = 712.47 (7) Å^3^
*M**_r_* = 659.41	*Z* = 1
Triclinic, *P*1	*F*(000) = 342
Hall symbol: -P 1	*D*_x_ = 1.537 Mg m^−^^3^
*a* = 6.8080 (4) Å	Mo *K*α radiation, λ = 0.71073 Å
*b* = 7.5569 (4) Å	θ = 1.4–27.0°
*c* = 14.3902 (8) Å	µ = 0.88 mm^−^^1^
α = 98.514 (4)°	*T* = 200 K
β = 92.062 (5)°	Prism, red
γ = 102.698 (5)°	0.12 × 0.08 × 0.04 mm

### Data collection

**Table:**

Stoe IPDS-1 diffractometer	2539 independent reflections
Radiation source: fine-focus sealed tube, Stoe IPDS-1	2539 reflections with *I* > 2σ(*I*)
Graphite monochromator	*R*_int_ = 0.033
φ scans	θ_max_ = 27.0°, θ_min_ = 1.4°
Absorption correction: numerical (*X-SHAPE* and *X-RED32*; Stoe & Cie, 2008)	*h* = −8→8
*T*_min_ = 0.800, *T*_max_ = 0.936	*k* = −9→9
3117 measured reflections	*l* = −18→16

### Refinement

**Table:**

Refinement on *F*^2^	Primary atom site location: structure-invariant direct methods
Least-squares matrix: full	Secondary atom site location: difference Fourier map
*R*[*F*^2^ > 2σ(*F*^2^)] = 0.030	Hydrogen site location: inferred from neighbouring sites
*wR*(*F*^2^) = 0.080	H atoms treated by a mixture of independent and constrained refinement
*S* = 1.06	*w* = 1/[σ^2^(*F*_o_^2^) + (0.0482*P*)^2^ + 0.0121*P*] where *P* = (*F*_o_^2^ + 2*F*_c_^2^)/3
3117 reflections	(Δ/σ)_max_ < 0.001
206 parameters	Δρ_max_ = 0.33 e Å^−^^3^
0 restraints	Δρ_min_ = −0.19 e Å^−^^3^

### Special details

**Table:**

Geometry. All e.s.d.'s (except the e.s.d. in the dihedral angle between two l.s. planes) are estimated using the full covariance matrix. The cell e.s.d.'s are taken into account individually in the estimation of e.s.d.'s in distances, angles and torsion angles; correlations between e.s.d.'s in cell parameters are only used when they are defined by crystal symmetry. An approximate (isotropic) treatment of cell e.s.d.'s is used for estimating e.s.d.'s involving l.s. planes.
Refinement. Refinement of *F*^2^ against ALL reflections. The weighted *R*-factor *wR* and goodness of fit *S* are based on *F*^2^, conventional *R*-factors *R* are based on *F*, with *F* set to zero for negative *F*^2^. The threshold expression of *F*^2^ > σ(*F*^2^) is used only for calculating *R*-factors(gt) *etc*. and is not relevant to the choice of reflections for refinement. *R*-factors based on *F*^2^ are statistically about twice as large as those based on *F*, and *R*- factors based on ALL data will be even larger.

### Fractional atomic coordinates and isotropic or equivalent isotropic displacement parameters (Å^2^)

**Table:**

	*x*	*y*	*z*	*U*_iso_*/*U*_eq_	
Ni1	0.5000	1.0000	0.0000	0.02742 (11)	
C1	0.1523 (3)	0.8520 (2)	0.32101 (13)	0.0331 (4)	
C2	0.3262 (3)	0.8457 (3)	0.37156 (15)	0.0435 (5)	
H2	0.4537	0.8789	0.3463	0.052*	
C3	0.3138 (4)	0.7905 (3)	0.45971 (16)	0.0516 (6)	
H3	0.4338	0.7874	0.4946	0.062*	
C4	0.1309 (5)	0.7404 (4)	0.49672 (18)	0.0651 (7)	
H4	0.1233	0.7025	0.5568	0.078*	
C5	−0.0420 (5)	0.7461 (5)	0.4453 (2)	0.0808 (10)	
H5	−0.1697	0.7112	0.4701	0.097*	
C6	−0.0314 (4)	0.8019 (4)	0.35813 (18)	0.0580 (6)	
H6	−0.1516	0.8058	0.3236	0.070*	
N1	0.1511 (3)	0.9162 (2)	0.23399 (12)	0.0329 (3)	
H1N1	0.056 (4)	0.957 (3)	0.2191 (18)	0.051 (7)*	
C7	0.2813 (3)	0.9123 (2)	0.16455 (13)	0.0281 (4)	
S1	0.21900 (7)	1.00329 (7)	0.06685 (3)	0.03549 (13)	
N2	0.4377 (2)	0.8401 (2)	0.17166 (11)	0.0312 (3)	
N3	0.5427 (2)	0.8441 (2)	0.08926 (11)	0.0300 (3)	
C8	0.6613 (3)	0.7309 (3)	0.07729 (14)	0.0337 (4)	
H8	0.739 (3)	0.737 (3)	0.0244 (16)	0.035 (5)*	
C9	0.6955 (3)	0.5875 (2)	0.12971 (13)	0.0317 (4)	
C10	0.8662 (3)	0.5206 (3)	0.10650 (14)	0.0364 (4)	
H10	0.9538	0.5760	0.0637	0.044*	
C11	0.9096 (3)	0.3751 (3)	0.14491 (14)	0.0362 (4)	
H11	1.0260	0.3307	0.1286	0.043*	
C12	0.7829 (3)	0.2951 (2)	0.20690 (14)	0.0322 (4)	
C13	0.6150 (3)	0.3636 (2)	0.23340 (13)	0.0302 (4)	
C14	0.5702 (3)	0.5083 (2)	0.19495 (13)	0.0315 (4)	
H14	0.4552	0.5539	0.2126	0.038*	
O1	0.8234 (2)	0.14835 (19)	0.24410 (11)	0.0411 (3)	
H1O1	0.7174	0.0896	0.2635	0.062*	
O2	0.5064 (2)	0.27403 (18)	0.29749 (10)	0.0392 (3)	
C15	0.3366 (4)	0.3391 (3)	0.33051 (18)	0.0478 (5)	
H15A	0.3816	0.4655	0.3637	0.072*	
H15B	0.2688	0.2604	0.3736	0.072*	
H15C	0.2423	0.3362	0.2769	0.072*	

### Atomic displacement parameters (Å^2^)

**Table:**

	*U*^11^	*U*^22^	*U*^33^	*U*^12^	*U*^13^	*U*^23^
Ni1	0.03166 (19)	0.03038 (17)	0.02632 (18)	0.01380 (13)	0.00733 (13)	0.01269 (13)
C1	0.0424 (11)	0.0294 (8)	0.0300 (10)	0.0098 (8)	0.0091 (8)	0.0086 (7)
C2	0.0491 (12)	0.0518 (12)	0.0369 (11)	0.0196 (10)	0.0086 (9)	0.0170 (9)
C3	0.0757 (17)	0.0529 (12)	0.0328 (11)	0.0258 (12)	0.0006 (11)	0.0125 (10)
C4	0.094 (2)	0.0699 (16)	0.0338 (12)	0.0125 (15)	0.0135 (14)	0.0237 (12)
C5	0.073 (2)	0.120 (3)	0.0497 (16)	0.0017 (18)	0.0235 (15)	0.0408 (17)
C6	0.0508 (14)	0.0816 (17)	0.0419 (13)	0.0049 (12)	0.0113 (11)	0.0246 (12)
N1	0.0338 (8)	0.0393 (8)	0.0330 (8)	0.0168 (7)	0.0097 (7)	0.0155 (7)
C7	0.0309 (9)	0.0265 (8)	0.0288 (9)	0.0073 (7)	0.0057 (7)	0.0092 (7)
S1	0.0338 (3)	0.0491 (3)	0.0333 (3)	0.0198 (2)	0.0090 (2)	0.0209 (2)
N2	0.0375 (8)	0.0353 (8)	0.0285 (8)	0.0173 (7)	0.0103 (7)	0.0140 (6)
N3	0.0349 (8)	0.0322 (7)	0.0281 (8)	0.0136 (6)	0.0083 (6)	0.0114 (6)
C8	0.0405 (10)	0.0369 (9)	0.0320 (10)	0.0188 (8)	0.0123 (8)	0.0149 (8)
C9	0.0377 (10)	0.0332 (9)	0.0302 (9)	0.0166 (8)	0.0069 (8)	0.0106 (7)
C10	0.0432 (11)	0.0371 (9)	0.0360 (10)	0.0180 (8)	0.0142 (9)	0.0127 (8)
C11	0.0366 (10)	0.0402 (10)	0.0393 (11)	0.0204 (8)	0.0094 (8)	0.0118 (8)
C12	0.0360 (10)	0.0306 (8)	0.0347 (10)	0.0137 (7)	0.0011 (8)	0.0114 (7)
C13	0.0334 (9)	0.0293 (8)	0.0303 (9)	0.0091 (7)	0.0043 (8)	0.0096 (7)
C14	0.0343 (9)	0.0308 (8)	0.0351 (10)	0.0152 (7)	0.0067 (8)	0.0112 (7)
O1	0.0414 (8)	0.0405 (7)	0.0524 (9)	0.0213 (6)	0.0094 (7)	0.0239 (6)
O2	0.0418 (8)	0.0387 (7)	0.0475 (8)	0.0176 (6)	0.0163 (7)	0.0240 (6)
C15	0.0532 (13)	0.0436 (11)	0.0576 (14)	0.0213 (10)	0.0292 (11)	0.0226 (10)

### Geometric parameters (Å, º)

**Table:**

Ni1—N3	1.9220 (15)	N2—N3	1.407 (2)
Ni1—N3^i^	1.9220 (15)	N3—C8	1.298 (2)
Ni1—S1^i^	2.1753 (5)	C8—C9	1.462 (2)
Ni1—S1	2.1753 (5)	C8—H8	0.94 (2)
C1—C6	1.376 (3)	C9—C10	1.398 (3)
C1—C2	1.380 (3)	C9—C14	1.402 (3)
C1—N1	1.409 (2)	C10—C11	1.383 (3)
C2—C3	1.392 (3)	C10—H10	0.9500
C2—H2	0.9500	C11—C12	1.375 (3)
C3—C4	1.370 (4)	C11—H11	0.9500
C3—H3	0.9500	C12—O1	1.375 (2)
C4—C5	1.380 (5)	C12—C13	1.398 (3)
C4—H4	0.9500	C13—O2	1.367 (2)
C5—C6	1.381 (4)	C13—C14	1.381 (2)
C5—H5	0.9500	C14—H14	0.9500
C6—H6	0.9500	O1—H1O1	0.8400
N1—C7	1.361 (2)	O2—C15	1.423 (2)
N1—H1N1	0.81 (3)	C15—H15A	0.9800
C7—N2	1.306 (2)	C15—H15B	0.9800
C7—S1	1.7322 (18)	C15—H15C	0.9800
			
N3—Ni1—N3^i^	180.00 (6)	C8—N3—N2	115.09 (15)
N3—Ni1—S1^i^	95.42 (5)	C8—N3—Ni1	123.66 (13)
N3^i^—Ni1—S1^i^	84.58 (5)	N2—N3—Ni1	121.20 (11)
N3—Ni1—S1	84.58 (5)	N3—C8—C9	131.97 (17)
N3^i^—Ni1—S1	95.42 (5)	N3—C8—H8	116.2 (13)
S1^i^—Ni1—S1	180.00 (3)	C9—C8—H8	111.8 (13)
C6—C1—C2	119.5 (2)	C10—C9—C14	119.10 (16)
C6—C1—N1	116.8 (2)	C10—C9—C8	114.36 (17)
C2—C1—N1	123.65 (18)	C14—C9—C8	126.46 (16)
C1—C2—C3	119.7 (2)	C11—C10—C9	120.99 (18)
C1—C2—H2	120.1	C11—C10—H10	119.5
C3—C2—H2	120.1	C9—C10—H10	119.5
C4—C3—C2	120.9 (2)	C12—C11—C10	119.49 (17)
C4—C3—H3	119.6	C12—C11—H11	120.3
C2—C3—H3	119.6	C10—C11—H11	120.3
C3—C4—C5	118.9 (2)	C11—C12—O1	119.75 (16)
C3—C4—H4	120.5	C11—C12—C13	120.43 (16)
C5—C4—H4	120.5	O1—C12—C13	119.81 (17)
C4—C5—C6	120.7 (3)	O2—C13—C14	125.69 (16)
C4—C5—H5	119.6	O2—C13—C12	113.95 (15)
C6—C5—H5	119.6	C14—C13—C12	120.35 (17)
C1—C6—C5	120.3 (3)	C13—C14—C9	119.58 (16)
C1—C6—H6	119.8	C13—C14—H14	120.2
C5—C6—H6	119.8	C9—C14—H14	120.2
C7—N1—C1	130.38 (17)	C12—O1—H1O1	109.5
C7—N1—H1N1	111.6 (19)	C13—O2—C15	117.54 (14)
C1—N1—H1N1	117.9 (19)	O2—C15—H15A	109.5
N2—C7—N1	121.37 (16)	O2—C15—H15B	109.5
N2—C7—S1	123.56 (14)	H15A—C15—H15B	109.5
N1—C7—S1	115.05 (13)	O2—C15—H15C	109.5
C7—S1—Ni1	96.21 (6)	H15A—C15—H15C	109.5
C7—N2—N3	110.66 (14)	H15B—C15—H15C	109.5

Symmetry code: (i) −*x*+1, −*y*+2, −*z*.

### Hydrogen-bond geometry (Å, º)

**Table:**

*D*—H···*A*	*D*—H	H···*A*	*D*···*A*	*D*—H···*A*
N1—H1*N*1···O1^ii^	0.81 (3)	2.37 (3)	3.122 (2)	154 (2)
O1—H1*O*1···N2^iii^	0.84	2.54	3.131 (2)	129

Symmetry codes: (ii) *x*−1, *y*+1, *z*; (iii) *x*, *y*−1, *z*.
